# Expression of GLUT3 and HIF-1*α* in Meningiomas of Various Grades Correlated with Peritumoral Brain Edema

**DOI:** 10.1155/2020/1682352

**Published:** 2020-08-29

**Authors:** Tao Mei, Zhengjun Wang, Jianwu Wu, Xianhua Liu, Wei Tao, Shousen Wang, Fuyong Chen

**Affiliations:** ^1^Department of Neurosurgery, Shenzhen University General Hospital, Shenzhen, Guangdong, China; ^2^Department of Neurosurgery, Fuzhou General Hospital, Fujian Medical University, Fuzhou, Fujian, China; ^3^Department of Gastroenterology, Fuzhou General Hospital, Fujian Medical University, Fuzhou, Fujian, China; ^4^Department of Pathology, Fujian Provincial Maternity and Children's Hospital, Fuzhou, Fujian, China; ^5^Clinical Research Center for Neurological Disorders, Shenzhen University, Shenzhen, Guangdong, China

## Abstract

**Aim:**

To investigate the expression of glucose transporter 3 (GLUT3) and hypoxia-inducible factor-1*α* protein (HIF-1*α*) in meningiomas and analyze the correlation between GLUT3 and HIF-1*α* expression with the pathological grade of peritumoral brain edema (PTBE) of meningiomas.

**Methods:**

In this cross-sectional study, we analyzed meningioma specimens from 160 patients collected from January 1, 2014, to December 1, 2017, by dividing them into a low-grade (WHO I) or high-grade (WHO II and WHO III) group. Immunohistochemical analyses were used to detect the expression level of GLUT3 and HIF-1*α* in the tumor specimens.

**Results:**

The proportion of GLUT3-positive staining in tumors sized <4 cm, 4–6 cm, and>6 cm was 35.9% (37/103), 63.6% (28/44), and 53.8% (7/13), respectively (*P* = 0.007). The proportion of HIF-1*α*-positive staining in tumors sized <4 cm, 4–6 cm, and >6 cm was 41.7% (43/103), 68.2% (30/44), and 38.5% (5/13), respectively (*P* = 0.010). The proportion of GLUT3-positive staining in the high-grade group and low-grade group was 70.8% (34/48) and 33.9% (38/112), respectively (*P* < 0.001). The proportion of HIF-1*α*-positive staining in the high-grade group and low-grade group was 62.5% (30/48) and 42.9% (48/112), respectively (*P* = 0.023). GLUT3-positive expression in meningioma PTBE grades 0, I, II, and III was 20.3% (13/64), 41.2% (14/34), 63.6% (21/33), and 82.8% (24/29), respectively (Bonferroni-corrected, *P* < 0.001, *α*/6 = 0.008). HIF-1*α*-positive expression in meningioma PTBE grades 0, I, II, and III was 34.4% (22/64), 47.1% (16/34), 54.5% (18/33), and 75.9% (22/29), respectively (Bonferroni-corrected, *P* = 0.003, *α*/6 = 0.008). Spearman's correlation analysis revealed a correlation between the expression of GLUT3 and HIF-1*α* in meningiomas (*r* = 0.463, *P* < 0.001). Multivariate analysis revealed that GLUT3-positive expression, HIF-1*α*-positive expression, and high pathological grade were associated with the development of PTBE (*P* < 0.05).

**Conclusions:**

GLUT3 and HIF-1*α* expression in meningiomas was closely related to the tumor size, pathological grade, and PTBE. This study is the first to report a unique map-like multifocal GLUT3 staining pattern in meningiomas.

## 1. Introduction

Glucose is the main energy substrate for most eukaryotic cells. Transmembrane transport of glucose into cells is a critical rate-limiting step in the metabolism of glucose. Transmembrane transport of glucose is facilitated by a family of proteins called glucose transporters (GLUTs). There are currently 14 known members of this family, of which GLUT3 plays a major role in glucose uptake in the brain [1]. In the case of hypoxic-ischemic encephalopathy, neurons can upregulate the expression of the GLUT family members, especially GLUT3 to increase tolerance to ischemia and hypoxia enabling the cells to survive and proliferate under these conditions [2]. Hypoxia-inducible factor-1*α* (HIF-1*α*) plays a crucial role in the development of the tumor [3]. HIF-1*α* is the most important regulator of GLUT3 [4], and it can promote the “Warburg effect” where tumor cells are guaranteed a sufficient supply of energy substances under hypoxic and ischemic conditions.

Meningiomas are among the most common types of intracranial tumors. Approximately 20–25% of meningiomas are WHO grade II, and 1–6% of meningiomas are grade III. High-grade meningiomas have significantly higher recurrence rates and a shorter survival time than benign WHO grade I meningiomas [5]. Peritumoral brain edema (PTBE) is a common symptom of meningioma. Severe PTBE increases the space the tumor occupies, aggravates the clinical symptoms, increases the difficulty of surgical resection, increases the severity of postoperative complications, prolongs postoperative treatment time; thus, it is an inherent challenge for clinical treatment [6].

Previous studies have shown an association of HIF-1*α* expression with different degrees of meningioma and perihematomal edema [7], and that hypoxia significantly increases glucose uptake and utilization in the tumor cells [8]. In this study, we investigated whether the expression of GLUT3 and HIF-1*α* in meningiomas is related to the tumor size, Ki-67, PTBE, and pathological grade. Exploring the changes in glucose metabolism in a hypoxic tumor microenvironment lays the foundation for further understanding of the underlying energy metabolism in meningiomas and provides a valuable reference for the precise treatment of meningiomas in the future.

## 2. Methods

### 2.1. Samples

A total of 160 surgically resected, pathologically confirmed meningioma samples were retrospectively collected from January 1, 2014, to December 1, 2017, at the Department of Neurosurgery of The Fuzhou General Hospital. All cases were diagnosed based on the analysis of complete medical records, images, and histopathology. The pathology department of Fuzhou General Hospital tested all tumors for Ki-67. Patients provided written informed consent for tissue and clinical data collection. This study was approved by the ethics review board of The Fuzhou General Hospital.

**Figure 1:**
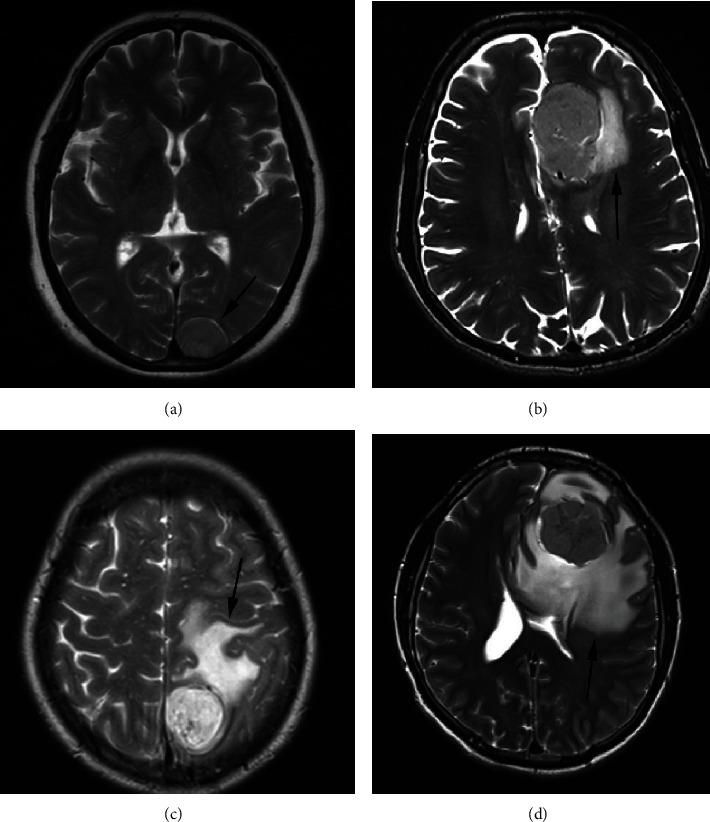
Types of peritumoral brain edema (PTBE) in meningiomas (black arrows). (a) No peritumoral edema around the meningioma, corresponding to PTBE grade 0. (b) Peritumoral edema (black arrow) around the tumor, but the edema range did not exceed 2 cm, corresponding to PTBE grade I. (c) Peritumoral edema (black arrow) around the tumor. The edema range is more than 2 cm but is limited to one half of the cerebral hemisphere, corresponding to PTBE grade II. (d) A wide peritumoral edema (black arrow) visible around the tumor, and the edema covers more than half of the cerebral hemisphere, corresponding to PTBE grade III.

All postoperative tumor specimens were graded by neuropathologists according to the World Health Organization (WHO) 2016 classification. As the WHO 2016 classification has not modified the classification scheme of 15 subtypes of meningioma nor warranted the assessment of specific molecular markers for the identification of subtype or grading [9], we divided the specimens into low-grade (WHO grade I) meningiomas and high-grade (WHO grade II or WHO grade III) meningiomas [10]. PTBE assessment was performed using magnetic resonance imaging, and the degree of PTBE of meningiomas was graded using the Steinhoff grading [7] according to the following scale: 0, no signs of edema; I, edema limited to 2 cm; II, edema limited to half of the hemisphere; and III, edema extending over more than half of the hemisphere (Figure 1). The values of Ki-67 proliferation index were divided into two groups: Group I ≤ 4% and Group II > 4% [11]. The tumor size was divided into three groups: Group 1 < 4 cm, Group 2 4–6 cm, and Group 3 > 6 cm [12].

### 2.2. Immunohistochemical Analysis

Rabbit anti-human polyclonal GLUT3 antibodies and Mouse anti-human monoclonal HIF-1*α* antibodies were purchased from Abcam (Cambridge, MA, USA).

EliVision immunohistochemistry was performed using a two-step method. All specimens were fixed in neutral formalin and embedded in paraffin. Serial sections were sliced at 4 *μ*m, routinely dewaxed, hydrated, and microwaved in 0.01 mol/L citrate buffer (pH 6.0) for 15 minutes. Endogenous peroxidase was inhibited by incubation for 10 minutes in 3% H_2_O_2_ and washed in PBS. The sections were blocked with 10% normal goat serum for 20 minutes at room temperature (25°C) and were not washed. GLUT3 antibody diluted at 1 : 400 and the HIF-1*α* antibody diluted at 1 : 100 were added dropwise, incubated overnight at 4°C, and washed in PBS. Sections were then incubated for 30 minutes with the secondary antibody at 37°C and then washed in PBS. The sections were stained with DAB, counterstained with hematoxylin, dehydrated, and sealed.

Positive controls were used according to the manufacturer's recommendation for the primary antibody protocol. PBS, instead of the primary antibody, was used as a negative control. Stained specimens were viewed using a CX41 microscope (Olympus, Tokyo, Japan) and imaged using Image Pro Plus 6.0 (Media Cybernetics, Rockville, MD, USA). The expression of GLUT3 is predominantly membranous or cytoplasmic [13]. The expression of HIF-1*α* is predominantly nuclear [14]. A zone in each section was identified under a low magnification microscope, wherein there was a uniform distribution of cells, and a high proportion of positive cells was observed under high power (200×). Serious local hypoxic conditions may eventually lead to necrosis; therefore, to determine the tumor hypoxia levels for statistical analysis, an estimate of the area of tumor necrosis was added to the estimate of the degree of GLUT3 staining [15].

A semiquantitative scoring system was used for the evaluation of GLUT3 expression that was based on both the staining intensity (0, none; 1, low; 2, moderate; 3, high) and percentage of positive cells (0, 0% cells; 1, 25% positive cells; 2, 26–50% positive cells; 3, 50% positive cells). The total score of protein expression in the tumor tissue in each case was the sum of these two scores; where a total score > 2 indicated positive expression and ≤2 indicated negative expression [16]. For evaluation of HIF-1*α* expression, the number of nonpositive cells or positive cells was less than 1%, of which 1–10% of positive cells had low expression (+), 11–50% had medium expression (++), and more than 50% had high expression (+++). A staining result of 0 or 1+ was considered as negative and ≥2+ as positive [17].

### 2.3. Statistical Analysis

All data were analyzed with SPSS Statistics for Windows, Version 23 (SPSS Inc., Chicago, IL, USA). Categorical variables were expressed in terms of absolute frequencies and percentages. Mean values were used to express the continuous variables. The two-variable correlation was analyzed using the Spearman's rank correlation with *a* = 0.05 as the test level. Data were analyzed using the *χ*^2^ test to assess the statistical difference. Bonferroni correction was used for multiple comparisons, and the *P* values were compared against *α*/number of comparisons instead of *α* = 0.05. Subsequently, multivariate logistic regression analysis was used to determine the factors independently associated with PTBE. The odds ratio (OR) for developing PTBE related to the variable was calculated. A value of *P* < 0.05 was considered statistically significant.

## 3. Results

The patient cohort consisted of 102 female and 58 male patients, with a female to male ratio of 1.76 : 1. The age of the patients ranged from 18 to 79 years with an average age of 53.08 ± 11.32 years, 102 patients were aged ≤60 years old and 58 > 60 years old. After reviewing the samples using the WHO classification, 112 cases appeared to be low-grade meningioma, including 31 fibrous, 41 transitional, 29 meningothelial, 6 psammomatous, 4 angiomatous, and 1 microcystic case, and 48 cases appeared to be high-grade meningioma, including 45 atypical, 1 chordoid, 1 rhabdoid, and 1 anaplastic case. PTBE was observed in 96 (67.2%) cases, which included 34 grade I, 33 grade II, and 29 grade III cases.

### 3.1. Expression Characteristics of GLUT3 and HIF-1*α* in Meningiomas

**Figure 2:**
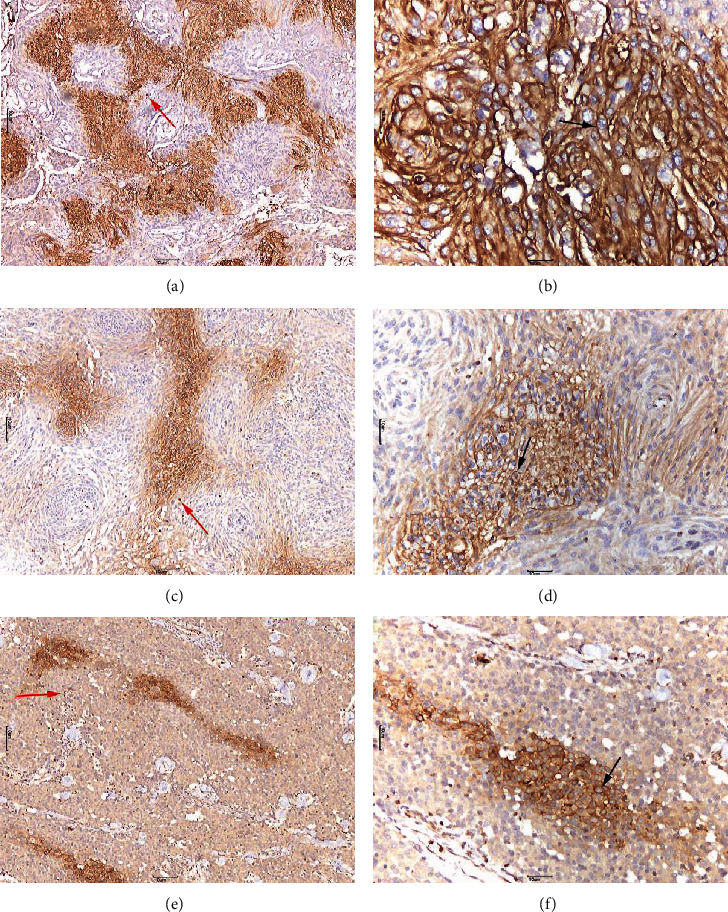
Glucose transporter 3 (GLUT3) membrane immunostaining of meningioma cells. (a, b) High GLUT3 staining ((a) 40×; (b) 200×), GLUT3 in meningioma cells shows “map-like” multifocal staining with strong positive expression. (c, d) Moderate GLUT3 staining ((c) 40×; (d) 200×). (e, f) Low GLUT3 staining ((e) 40×; (f) 200×). Membrane-positive staining (black arrows) is evident in tumor cells. Cells with positive GLUT3 expression are separated from the intratumoral vascular network. Notably, we found GLUT3 expression in most lymphocytes ((a, c, and e) red arrows). (a–d) are regarded as positive results, whereas (e, f) are regarded as negative results.

**Figure 3:**
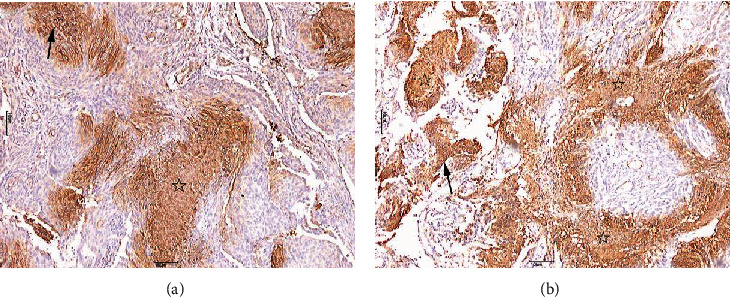
Meningioma characterized by a “map-like” multifocal staining with small- ((a) 40×) and large- ((b) 40×) sized areas of necrosis with cell detritus, indicated by asterisks. Glucose transporter 3-positive staining of meningioma cells (black arrows).

**Figure 4:**
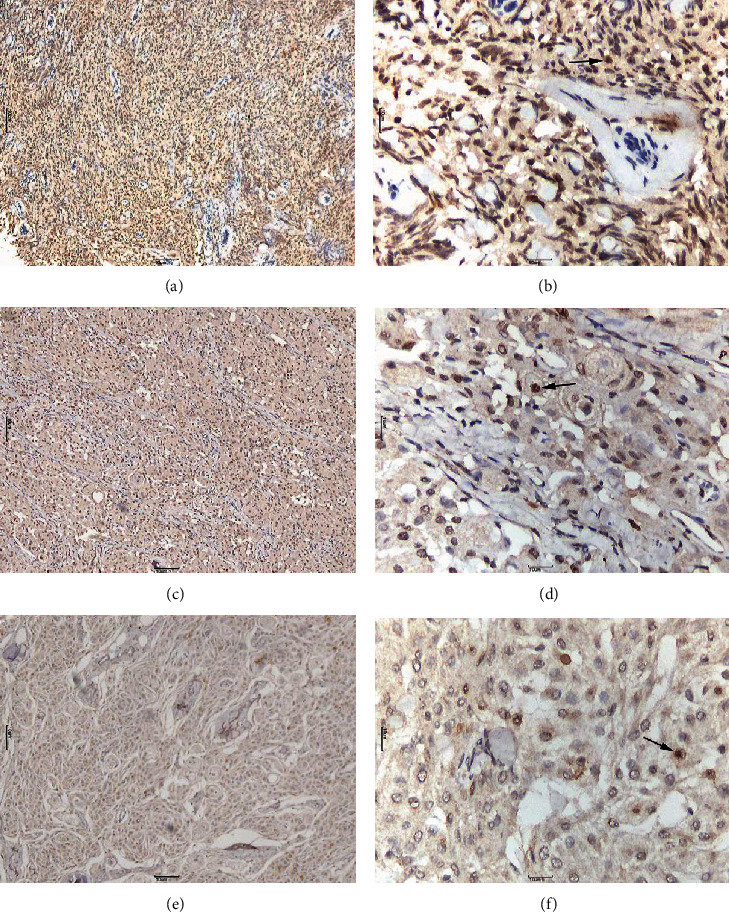
Hypoxia-inducible factor-1*α* protein (HIF-1*α*) nuclear immunostaining of meningioma cells. (a, b) High HIF-1*α* staining ((a) 40×; (b) 200×); HIF-1*α* strongly and diffusely expressed in meningioma cells (×40). (c, d) Moderate HIF-1*α* staining ((c) 40×; (d) 200×). (e, f) Low HIF-1*α* staining ((e) 40×; (f) 200×). Nucleus positive staining (black arrows) is evident in tumor cells. (a–d) are regarded as positive results, whereas (e, f) are regarded as negative results.

**Figure 5:**
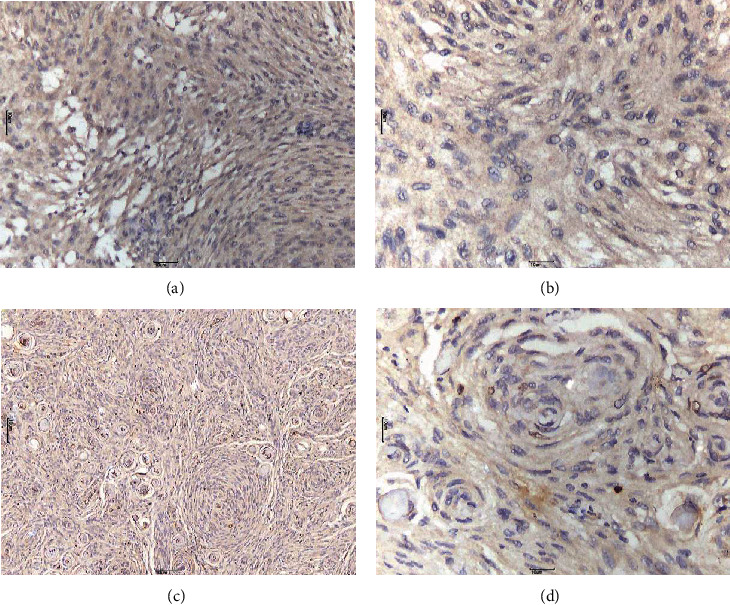
Meningioma cells without glucose transporter 3 immunostaining ((a) 40×; (b) 200×) and HIF-1*α* immunostaining ((c) 40×; (d) 200×).

GLUT3 positive expression was mainly characterized by granular brown staining in the cell membrane or the cytoplasm. Owing to the biological significance of membrane localization of GLUT3, only cell membrane staining was studied. GLUT3 showed a “map-like” multifocal staining pattern (Figures 2 and 3). Cells with positive GLUT3 expression were separated from the intratumoral vascular network. Notably, GLUT3 immune expression was also observed in lymphocytes. HIF-1*α* expression was mainly characterized by brown staining of the nucleus (Figure 4). There were also some tumor cells that did not show GLUT3 or HIF-1*α* staining (Figure 5).

### 3.2. Relationship between GLUT3 and HIF-1*α* Expression with Pathological Grade, Tumor Size, and Ki-67

Among the 112 cases of low-grade meningiomas, GLUT3 positive expression was observed in 38 (33.9%), whereas among the 48 cases of high-grade meningiomas, GLUT3 positive expression was observed in 34 (70.8%). This finding was statistically significant (*P* < 0.001) (Table 1). Of the 112 low-grade meningiomas, 48 (42.9%) exhibited a high HIF-1*α* expression, whereas of the 48 high-grade meningiomas, 30 (62.5%) exhibited a high HIF-1*α* expression, which was also statistically significant (*P* = 0.023) (Table 1).

We found a significant difference in the expression of GLUT3 between tumor sizes of <4 cm, 4–6 cm, and >6 cm, with a proportion of 35.9% (37/103), 63.6% (28/44), and 53.8% (7/13), respectively (*χ*^2^ = 10.015, *P* = 0.007) (Table 1). From the pairwise comparisons, we found that there was a statistically significant difference between <4 cm and 4–6 cm (Bonferroni-corrected *P* = 0.002). Additionally, there was a significant difference in the expression of HIF-1*α* between tumor sizes of <4 cm, 4–6 cm, and >6 cm, with a proportion of 41.7% (43/103), 68.2% (30/44), and 38.5% (5/13), respectively (*χ*^2^ = 9.222, *P* = 0.010) (Table 1). From the pairwise comparisons, we found that there was a statistically significant difference between <4 cm and 4–6 cm (Bonferroni-corrected *P* = 0.003) (Table 1).

### 3.3. Relationship of PTBE with GLUT3 and HIF-1*α* Expression in Meningiomas

We found statistically significant differences in GLUT3-positive staining, with a frequency of 20.3% in grade 0 (13/64), 41.2% in grade I (14/34), 63.6% in grade II (21/33), and 82.8% in grade III (24/29) PTBE in our cohort (*χ*^2^ = 37.297, *P* < 0.001, *α*/6 = 0.008). GLUT3 expression increased with increased peritumoral edema (Table 2). We also found significant differences in HIF-1*α*-positive staining, with a frequency of 34.4% in grade 0 (22/64), 47.1% in grade I (16/34), 54.5% in grade II (18/33), and 75.9% in grade III (22/29) in our cohort (*χ*^2^ = 14.308, *P* = 0.003, *α*/6 = 0.008). HIF-1*α* expression increased with increased peritumoral edema (Table 2).

### 3.4. Relationship between GLUT3 and HIF-1*α* Expression in Meningiomas

In this study, GLUT3 expression was compared with HIF-1*α* expression (Table 3), and a Spearman's rank correlation analysis was used to analyze the correlation between the two expression patterns. There was a significant positive correlation (*r* = 0.463, *P* < 0.001), with GLUT3 expression increasing with increased expression of HIF-1*α*.

### 3.5. Multivariate Analysis for PTBE

Analysis of the five factors potentially affecting PTBE by multivariate logistic regression (Table 4) revealed that GLUT3 positive staining (*P* < 0.001; OR: 11.267, 95% confidence interval [CI]: 4.923–25.789), HIF-1*α* positive staining (*P* = 0.006; OR: 2.949, 95% CI: 1.364–6.376), and high pathological grade (*P* = 0.023; OR: 2.775, 95% CI: 1.154-6.671) were associated with the development of PTBE (Table 4).

## 4. Discussion

Nishioka et al. investigated the expression of GLUT3 mRNA in meningiomas using in situ hybridization [18]. Glick et al. also demonstrated the expression of GLUT3 together with IGF-II mRNA in meningiomas [19]. In this study, we explored the expression of GLUT3 protein in meningiomas. We have discovered a unique “map-like” multifocal staining pattern that has not been previously described in other tumor types. This type of map-like staining is more regular than patchy. The results of this study indicate a statistically significant difference in the expression of GLUT3 and HIF-1*α* in meningiomas of different tumor sizes and pathological grades. In addition, there was a statistically significant difference in the pattern of expression of GLUT3 and HIF-1*α* between meningiomas with different severity of peritumoral edema. Therefore, GLUT3 may play an important role in the process of hypoxia-induced PTBE in meningiomas.

Nes et al. [15] have found that GLUT1 expression is spatially separated from the intratumoral vascular network, indicating that this atypical histologic alteration represents a cellular response to diffusion-limited hypoxia. GLUT3 is the most abundant glucose transporter in the brain except for GLUT1. However, its transport capacity is higher than that of GLUT1 [20]. Our study showed that cells with positive GLUT3 expression in meningiomas are also separated from the intratumoral vascular network. Differences in oxygen diffusion inside a tumor lead to different glucose metabolism dynamics across the separate regions. GLUT3 expression is separated from the blood vessels, most of it surrounds the blood vessels, especially in specimens with a strong positive expression. We also observed GLUT3 expression in most lymphocytes which is similar to Mochizuki et al. [21], who observed high expression of GLUT1 and GLUT3 in inflammatory tissues, alongside the malignant tumors. Rojas et al. [22] found that although the expression of GLUT1 in the tumor tissues was significantly higher than in the inflammatory tissues, GLUT3 expression was higher in the inflammatory lesions. This finding may reflect the involvement of GLUT3 in earlier stages of carcinogenesis in some tumors. Therefore, we hypothesize that GLUT3 staining of meningiomas may indicate a heightened sensitivity to hypoxia, as compared with GLUT1. Both GLUT1 and GLUT3 may play important roles in different growth stages of meningiomas, promoting each other, and improving the tolerance of meningiomas to hypoxia and ischemia at different stages. In addition, meningiomas with high levels of GLUT3 expression are more prone to necrosis. Tumor cells adapt to hypoxic microenvironment by upregulating GLUT3 to compensate for the increased demand for glucose, but severe local hypoxic conditions may eventually lead to cell death.

Studies have shown that HIF-1*α* expression is significantly different in meningiomas of different pathological grades and that HIF-1*α* expression is positively correlated to the malignancy of the meningioma [23]. We observed that GLUT3 and HIF-1*α* expression significantly increased with the size and pathological grade of the meningioma. As the tumor grows and its pathological grade increases, tumor cells are exposed to ischemia and hypoxia, conditions under which GLUT3 expression is more easily induced.

PTBE is a common, concomitant sign of meningioma. However, the mechanisms underlying the formation of PTBE are complicated. Most studies have shown that physical compression of tissues and the release of cytokines work together to cause PTBE, more specifically, angiogenic brain edema. The location and size of the tumor, venous reflux disorder, and secretion of various cytokines are all related to PTBE formation [24]. Tumor cells alter the permeability of blood vessels inside and outside by destroying the tight junctions of microvascular endothelial cells in the blood–brain barrier (BBB), thereby inducing PTBE [25]. Some studies have also shown that PTBE is positively correlated with the expression of HIF-1*α* in tumors, and that the higher the expression of HIF-1*α*, the more severe is the PTBE [7]. This may be because HIF-1*α* can induce high expression of various cytokines, GLUT, and vascular endothelial growth factor (VEGF), stimulate the formation of a corresponding vascular network, and change the permeability of the BBB. The induction of peritumoral edema is secondary to these effects. Recent research on meningioma PTBE has revealed that the release of cytokines secondary to hypoxia accounts for most cases of PTBE induction. Increased HIF-1*α* expression in meningiomas is not only known to activate VEGF expression but also triggers the other consequences of hypoxia in meningioma [26, 27].

In this study, the incidence of PTBE was 67.2% and statistical analysis showed that there were significant differences in the expression level of both GLUT3 and HIF-1*α* between groups with different degrees of PTBE. Meningioma with significant PTBE has higher GLUT3 expression levels, which may be related to GLUT3 being the main glucose transporter for nerve cells to absorb glucose. On one hand, tumor cells can induce neovascularization and increase blood supply. On the other hand, tumor cells can increase glucose uptake and maintain a high energy metabolism rate by increasing GLUT3 expression. Our study shows that the expression of GLUT3 differs across PTBE types, indicating that the degree of ischemia and hypoxia differs across the PTBE types. In addition to increasing the blood supply to take in more oxygen, the cells are also able to acquire additional energy through the upregulation of GLUT3. Kan et al. [28] observed a correlation between the meningioma grade and peritumoral edema, with high-grade meningiomas being more prone to developing PTBE. Our findings completely agree with these previous findings. The results of this study indicate that high-grade meningiomas are more likely to express GLUT3 and that meningiomas with PTBE have higher rates of glucose metabolism. The proliferation of tumor cells and an increase in glucose uptake suggests that meningiomas with PTBE may be invasive.

Our study had some limitations. In particular, due to the small number of cases with certain meningioma subtypes, we were not able to explore GLUT3 and HIF-1*α* expression patterns across all subtypes of WHO grade meningiomas.

## 5. Conclusions

The results of this study indicated that GLUT3 is highly expressed in meningiomas with PTBE. Further, this study revealed that meningiomas with PTBE may be more susceptible to ischemia and hypoxia. Finally, GLUT3 is highly expressed in high-grade meningiomas, and rapid growth of tumor cells may upregulate the expression of GLUT3 to meet the energy requirements for tumor growth.

## Figures and Tables

**Table 1:** Clinical characteristics of meningiomas with GLUT3 and HIF-1*α* expression.

Factors	*n*	GLUT3 staining		*χ* ^2^	*P*	HIF-1*α* staining		*χ* ^2^	*P*
		Positive	Negative			Positive	Negative		
Grade									
Low	112	38	74			48	64		
High	48	34	14	18.490	0.001	30	18	5.189	0.023
Ki-67									
>4	46	26	20			24	22		
≤4	114	46	68	3.463	0.063	54	60	0.303	0.582
Size									
<4 cm	103	37	66			43	60		
4–6 cm	44	28	16			30	14		
>6 cm	13	7	6	10.015	0.007	5	8	9.222	0.010

**Table 2:** GLUT3 and HIF-1*α* expression in correlation with the grade of PTBE.

PTBE	*n*	GLUT3 staining		*χ* ^2^	*P*	HIF-1*α* staining		*χ* ^2^	*P*
		Positive	Negative			Positive	Negative		
0	64	13	51			22	42		
I	34	14	20			16	18		
II	33	21	12			18	15		
III	29	24	5	37.297	<0.001	22	7	14.308	0.003

PTBE: peritumoral brain edema.

**Table 3:** Correlation between GLUT3 and HIF-1*α* protein expression in meningiomas.

GLUT3 staining	HIF-1*α* staining				*n*	*r*	*P*
	—	+	++	+++			
—	42	7	8	11	68		
+	6	6	3	5	20		
++	4	5	9	10	28		
+++	3	9	12	20	44	0.463	<0.001

^∗^Statistically significant differences (*P* < 0.001) when confidence level (bilateral) is 0.05.

**Table 4:** Multivariate analysis for PTBE.

Variable	Multivariate analysis	
	OR (95% CI)	*P*
Female sex	0.889 (0.426-1.855)	0.754
Aged ≤60 years	0.634 (0.296-1.359)	0.241
GLUT3 positive staining	11.267 (4.923-25.789)	<0.001
HIF-1*α* positive staining	2.949 (1.364-6.376)	0.006
High pathological grade	2.775 (1.154-6.671)	0.023

## Data Availability

The datasets used during the current study are available from the corresponding author on reasonable request.
